# Relative hematocrit declines at the time of transfusion and postoperative outcomes after coronary artery bypass grafting: a retrospective cohort study

**DOI:** 10.1016/j.bjane.2026.844813

**Published:** 2026-08-07

**Authors:** Fatemeh Sadr, Arash Jalali, Ahmad Vakili-Basir, Mina Pashang, Khosro Barkhordari

**Affiliations:** a Department of Anesthesiology and Critical Care, Tehran Heart Center, Tehran University of Medical Sciences, Tehran, Iran; b Tehran Heart Center, Cardiovascular Diseases Research Institute, Tehran University of Medical Sciences, Tehran, Iran; c Department of Epidemiology and Biostatistics, School of Public Health, Tehran University of Medical Sciences, Tehran, Iran

**Keywords:** Blood transfusion, Cardiac surgical procedures, Coronary artery bypass, Hematocrit, Postoperative complications

## Abstract

**Background:**

Despite advances in patient blood management, the optimal hemoglobin threshold for Red Blood Cell (RBC) transfusion remains controversial. This observational study evaluated the association between the relative decline in Hematocrit (HCT) at the time of transfusion and postoperative outcomes in patients undergoing Coronary Artery Bypass Graft (CABG) surgery.

**Methods:**

We conducted a retrospective cohort study of 629 patients who underwent CABG and received a transfusion at Tehran Heart Center Hospital, Iran, between March 2018 and December 2021. Based on the relative hematocrit reduction at the time of transfusion, patients were divided into two groups: Group A (≥ 40% change) and Group B (< 40% change). Early postoperative outcomes were compared between groups. Primary outcomes included mortality, hospital length of stay, intensive care unit length of stay, and intubation time. The composite secondary outcome comprised postoperative infection, renal failure, tamponade, and reoperation due to bleeding.

**Results:**

There were no significant differences in primary outcomes, except hospital length of stay (p = 0.02). However, after adjustment for potential confounders, this difference was no longer significant (p = 0.32). Composite secondary outcomes were comparable between the two groups, with no significant difference observed (p = 0.85).

**Conclusion:**

In patients undergoing CABG surgery, this study did not identify a significant difference in early postoperative outcomes between two transfused patient groups stratified by a 40% change in HCT. Further prospective studies are needed to determine clinical relevance of relative hematocrit decline at the time of transfusion as a potential adjunct to conventional transfusion triggers.

*THC institutional review board:* IR.TUMS.THC.REC.1402.070.

## Introduction

After extensive research in the field of blood transfusion, the ideal trigger for transfusion has still not been clearly defined. Some believe that blood transfusion based solely on a fixed threshold is not clinically desirable. Although restrictive transfusion strategies are widely endorsed, they may overlook the unique needs of certain patient populations.^1^ Therefore, some studies have suggested using the percentage change in hemoglobin levels instead of a fixed threshold. In fact, some studies in surgical patients showed that if the percentage change in hemoglobin was 50% or greater, the risk of complications, such as ischemic events, was higher.^2^ The primary objective of transfusion is improving oxygenation. Beyond general physiological responses to anemia, such as increased iron absorption, distribution, and mobilization, and organ adaptation, cellular adaptation plays a key role in chronic anemia.^3^ Alongside other cellular adaptation mechanisms in response to cellular hypoxemia, such as alterations in transcription, translation, and post-translational pathways, and modifications to metabolite-dependent activity, mitochondrial adaptation plays a major role in both acute and chronic cellular hypoxia.^4^ These regulatory mechanisms vary with the chronicity and severity of changes, thereby affecting cells' ability to respond adequately to various levels of stress, including hypoxia caused by anemia. Hypoxia-Induced Factor-1 (HIF-1α) is one example of an acute cellular adaptation that increases oxygen delivery and decreases oxygen consumption, whereas in chronic settings, HIF-2α is a major determinant of organ adaptation.^5^

Logically, a patient with a preoperative hemoglobin level of 17 g.dL^−1^ would require significant blood loss to reach a postoperative level of 7 g.dL^−1^. It does not seem prudent to assume that another patient with a baseline hemoglobin of 10 g.dL^−1^ would experience the same stress responses to anemia with a current post-bleeding hemoglobin of 7 g.dL^−1^. Several studies have evaluated clinical outcomes according to the magnitude of hemoglobin decline from baseline at the time of transfusion. In the study by Spolverato G and colleagues, mortality and ischemia-specific complications in patients undergoing major gastrointestinal surgery, as well as relative hemoglobin reduction, were evaluated. Patients with a relative hemoglobin reduction of > 50% whose nadir hemoglobin remained ≥ 7 g.dL^−1^ were more likely to develop postoperative complications.^6^ Another study reported that among transfused patients, outcomes worsened with a substantial hemoglobin decline, regardless of nadir hemoglobin levels.^7^ These findings raise questions regarding reliance solely on an absolute hematocrit threshold, prompting us to perform a retrospective study evaluating whether the percentage change in hematocrit observed at the time of transfusion was associated with early postoperative outcomes. The percentage change in Hematocrit (ΔHCT) was calculated using the formula: ΔHCT=[(baselineHCT−transfusion−timeHCT)/baselineHCT]×100.

We assessed whether postoperative outcomes differed by the magnitude of hematocrit decline at the time of packed red blood cell transfusion.

## Methods

This single-center, retrospective, observational study compared the early postoperative outcomes of patients who received packed red blood cells (PRBCs) and were subsequently divided into two groups based on the relative change in hematocrit levels (ΔHCT). The study population included all adult patients who underwent coronary artery bypass grafting (CABG) at Tehran Heart Center between March 2018 and December 2021, had documented pre- and postoperative hematocrit changes, and received blood transfusions. Data were collected by evaluating patients’ medical records, the hospital database, and intensive care unit (ICU) flow sheets. A total of 1,800 patient records were screened, of which 629 patients were included in the final analysis. The exclusion criteria included: age less than 18, history of pathological bleeding in the three previous months, recent myocardial infarction (MI), history of receiving blood in the past two weeks, blood dyscrasias, renal failure, and hepatic failure. All CABG procedures were included, including those combined with other cardiac operations (e.g., valve repair). Cases without CABG were excluded. To maintain analytical rigor and enhance patient homogeneity, off-pump patients were excluded from this study. This exclusion was required due to the limited number of cases in this subgroup and the significant confounding effects of cardiopulmonary bypass (CPB) on critical physiological parameters, including hemodynamics, coagulation, and hematocrit, which influence transfusion requirements. The primary objective was to evaluate early postoperative outcomes, including ICU length of stay, intubation time, 30-day in-hospital mortality, and total hospital length of stay. Secondary outcomes included the incidence of postoperative infections, atrial fibrillation (AF), acute kidney injury (AKI), postoperative encephalopathy, and reoperation due to bleeding. For the assessment of secondary outcomes, a composite endpoint was defined as the occurrence of postoperative infection, cardiac tamponade, reoperation due to bleeding, or renal failure.

**Figure 1:**
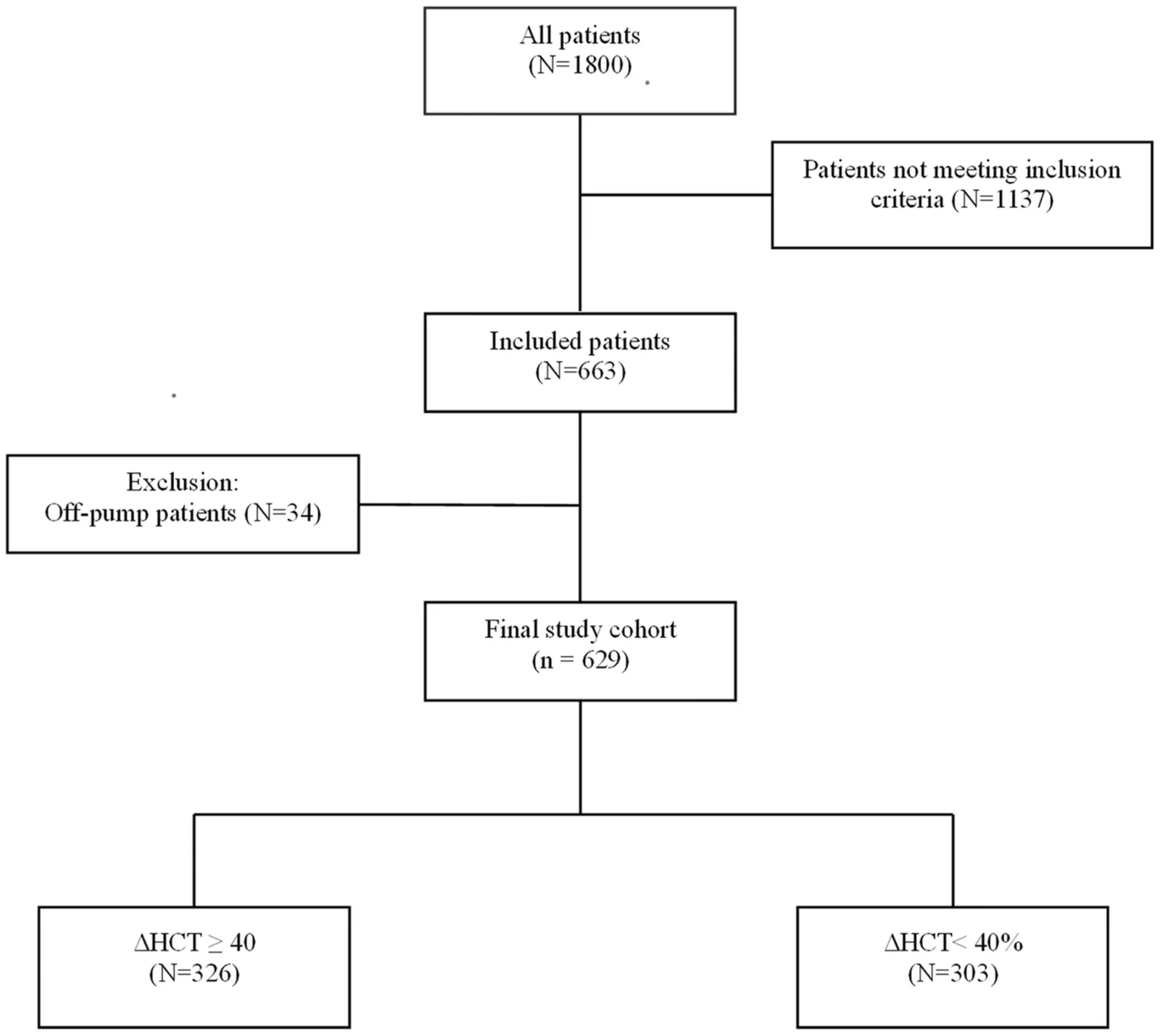
Flow diagram of patient selection for the study. Among 1,800 reviewed records, 663 coronary artery bypass grafting (CABG) patients met the inclusion criteria. After excluding 34 off-pump cases, 629 patients were included in the final analysis, with 326 experiencing relative hematocrit declines ≥ 40% and 303 with relative hematocrit declines < 40%.

Infections included pneumonia, mediastinitis, and wound infections. Acute kidney injury was defined according to the acute kidney injury network (AKIN) criteria as an abrupt decline in renal function occurring within 48 hours after surgery, identified by an increase in serum creatinine of ≥ 0.3 mg.dL^−1^, an increase to ≥ 1.5 times the baseline serum creatinine, or a urine output of < 0.5 mL.kg^−1^.h^−1^ for more than 6 hours.^8^ Postoperative renal failure was defined as renal failure diagnosed during the index hospitalization by the consulting nephrologist. In patients with suspected postoperative renal dysfunction, a nephrology consultation was requested, and the diagnosis was established based on the consulting nephrologist's clinical assessment. Postoperative encephalopathy was diagnosed by the hospital neurologist and encompassed patients who experienced delirium, coma, or seizures at any point during the three-day postoperative period. Figure 1 presents the patient flow diagram, categorized according to the established patient selection criteria.

Anesthesia was performed according to the standard protocol for CABG, and extubation was performed based on the established ICU protocol. The administration of PRBCs is typically guided by absolute hemoglobin levels and follows our hospital’s protocol, which aligns with the transfusion practices of most centers ‒ generally based on hemoglobin thresholds of 7–8 g.dL^−1^. However, this guideline was occasionally overridden at the discretion of the surgical team, depending on specific intraoperative or clinical considerations. We identified patients who received transfusions and subsequently evaluated the change in hematocrit at the time of transfusion. The baseline hematocrit was defined as the mean hematocrit within the 3 days preceding surgery. The hematocrit value at the time of PRBC transfusion ‒ whether in the operating room or the intensive care unit ‒ was documented as the transfusion-time HCT. The percentage change in hematocrit (ΔHCT) was calculated using the formula: (ΔHCT)=[(baselineHCT−transfusion−timeHCT)/baselineHCT]×100.

Based on the magnitude of the ΔHCT, we categorized the patients into two groups. The first group consisted of patients whose hematocrit had declined by 40% or more at the time of transfusion (group A), while the second group included those whose hematocrit had declined less than 40% at the time of transfusion (group B).

This study was reviewed and approved by the Ethics Committee of Tehran University of Medical Sciences (Approval n° IR.TUMS.THC.REC.1402.070). Written informed consent for the use of clinical data was obtained as part of the routine admission process at Tehran Heart Center.

### Statistical analysis

**Figure 2:**
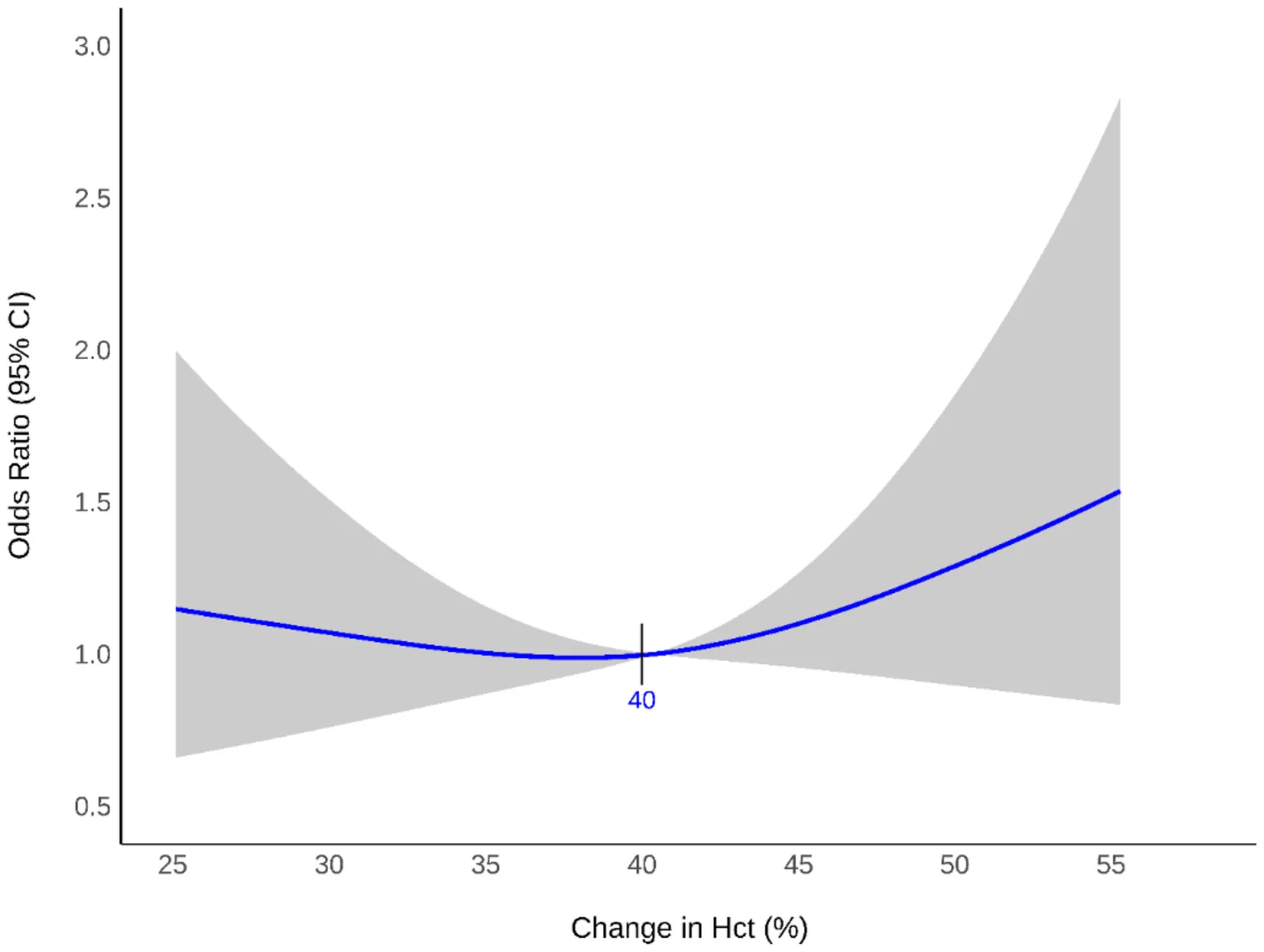
Restricted cubic spline (RCS) analysis showing the association between change in hematocrit (ΔHCT) and the odds of the study outcome. The solid blue line represents the estimated odds ratio, and the shaded area indicates the 95% confidence interval. A ΔHCT of 40% was used as the reference value (OR = 1.0). The spline suggests a nonlinear relationship, with increasing odds at higher ΔHCT levels.

Continuous variables are presented as mean ± SD (Standard Deviation) or median (Interquartile Range boundaries; IQR). Categorical variables are expressed as frequencies and percentages. Quantitative variables were compared using the Student’s *t*-test or the Mann‐Whitney *U*-test, as appropriate. In contrast, categorical variables were compared using the Chi-Squared or Fisher's exact test as required. A restricted cubic spline (RCS) with three knots at the 25^th^, 50^th^, and 75^th^ percentiles was employed to determine the turning point of the ΔHCT variable that changed its effect on secondary composite outcomes. Based on the optimum proposed cut-off, the ΔHCT variable was divided into two levels (Fig. 2 and Supplementary Fig. S1). Linear and logistic regression models, adjusted for literature-identified confounders using stabilized inverse probability weighting (sIPW), were applied to analyze ΔHCT levels. The standardized mean difference (SMD) served as the metric for assessing covariate balance through graphical representation in Supplementary Figure S2. The association between the exposure and primary outcomes [hospital length of stay (hospital LOS), intensive care unit length of stay (ICU LOS), and intubation time] and a composite secondary outcome (postoperative infection, renal failure, reoperation due to surgical bleeding, and tamponade) was examined using linear and logistic regression analyses. These models were adjusted using stabilized inverse probability weighting. The results were presented as beta and odds ratio (OR), with 95% confidence intervals (95% CIs). To evaluate the robustness of the findings to missing data, a sensitivity analysis was performed, and the results are presented in Supplementary Table S1. All p-values were two‐tailed, and a p < 0.05 was considered to be statistically significant. Analyses were conducted using the R Statistical language (version 4.3.0; R Core Team, 2023)^9^ using the packages Weight It (version 1.4.0; Greifer N, 2025)^10^ and plotRCS (version 0.1.5; Huo R, 2025).^11^^,^^12^

## Results

Initially, 1800 CABG patients were screened, of whom 663 met the study inclusion criteria. After the exclusion of 34 patients due to off-pump surgical procedures, 629 patients comprised the final analytical cohort. Within this cohort, 48% had a ΔHCT below 40%, whereas 52% had a ΔHCT of 40% or greater at the time of transfusion. The patient cohort exhibited a mean age of 63 ± 8 years. Baseline hematocrit levels averaged 38.8% ± 4.4%, while the mean HCT at the point of transfusion was 23.2% ± 3.4%. While age did not differ significantly between the groups (p = 0.60), statistically significant variations were evident in the latter two parameters. The average change in Hematocrit (ΔHCT) across the patient population was 15.65% ± 3.8%. Table 1 illustrates the basic characteristics and clinical data of the two groups.

**Table 1:** Baseline characteristics and perioperative variables according to relative HCT reduction in CABG patients.

Variables	Overall (n = 629)^a^	Relative HCT reduction		p-value^b^
		≥ 40 (n = 326)^a^	< 40 (n = 303)^a^	
Age, years	63.58 ± 8.51	63.83 ± 8.37	63.31 ± 8.66	0.604
Male sex, n (%)	377 (59.9%)	164 (50.3%)	213 (70.3%)	< 0.001
BMI, kg.m^−2^	27.47 ± 4.20	27.43 ± 4.22	27.51 ± 4.19	0.823
Diabetes Mellitus, n (%)	313 (49.8%)	177 (54.3%)	136 (44.9%)	0.018
Hypertension, n (%)	436 (69.3%)	237 (72.7%)	199 (65.7%)	0.056
Hyperlipidemia, n (%)	358 (56.9%)	199 (61.0%)	159 (52.5%)	0.030
Smoking, n (%)	233 (37.0%)	111 (34.0%)	122 (40.3%)	0.107
COPD, n (%)	36 (5.7%)	12 (3.7%)	24 (7.9%)	0.022
Pre-op HCT, %	38.86 ± 4.42	39.74 ± 4.22	37.92 ± 4.44	< 0.001
HCT before transfusion, %	23.21 ± 3.40	21.53 ± 2.72	25.02 ± 3.12	< 0.001
LVEF, %	50.0 (40.0, 55.0)	50.0 (40.0, 55.0)	45.0 (40.0, 55.0)	0.007
Baseline Creatinine, mg.dL^−1^	1.1 (0.9, 1.2)	1.1 (0.9, 1.2)	1.1 (0.9, 1.2)	0.794
EuroSCORE II	1.8 (1.2, 4.4)	2.0 (1.2, 5.1)	1.6 (1.1, 4.2)	0.056
CAG, n (%)				0.849
SVD	38 (6.0%)	20 (6.1%)	18 (5.9%)	
2VD	88 (14.0%)	48 (14.7%)	40 (13.2%)	
3VD	503 (80.0%)	258 (79.1%)	245 (80.9%)	
Operation, n (%)				0.785
Isolated CABG	495 (78.7%)	253 (77.6%)	242 (79.9%)	
CABG + Valve	55 (8.7%)	30 (9.2%)	25 (8.3%)	
CABG + Valve + Other	39 (6.2%)	23 (7.1%)	16 (5.3%)	
CABG + Other	40 (6.4%)	20 (6.1%)	20 (6.6%)	
Surgery, n (%)				0.384
Elective	583 (92.7%)	305 (93.6%)	278 (91.7%)	
Emergent/urgent	46 (7.3%)	21 (6.4%)	25 (8.3%)	
Cross Clamp Time, min	46.5 (37.0, 60.0)	47.0 (38.0, 60.0)	45.0 (36.0, 60.0)	0.223
CPB Time, min	83.0 (65.0, 110.0)	86.0 (70.0, 110.0)	80.0 (63.0, 110.0)	0.115
Post-op Systolic BP mean, mmHg^c^	134.1 ± 15.7	136.2 ± 15.2	137.2 ± 12	0.120
Post-op Diastolic BP, mmHg^c^	78 ± 4.3	79 ± 5.4	75 ± 8.3	0.113
Heparin, I/U	29,765.50 ± 7,557.60	29,438.65 ± 7,125.31	30,117.16 ± 7,993.60	0.213
Protamine, mg	350.0 (300.0, 400.0)	350.0 (300.0, 400.0)	400.0 (300.0, 400.0)	0.191
Transamin, gr	2.0 (2.0, 3.0)	2.0 (2.0, 3.0)	2.0 (2.0, 3.0)	0.649
Blood drainage, mL^d^	450.0 (350.0, 550.0)	450.0 (350.0, 550.0)	450.0 (350.0, 550.0)	0.427
PRBC, units^e^	2.0 (1.0, 3.0)	2.0 (1.0, 3.0)	2.0 (1.0, 3.0)	< 0.001
Urine in OR, mL	1,800 (1,500.0, 2,300.0)	1,900.0 (1,500.0, 2,300.0)	1,800.0 (1,350.0, 2,200.0)	0.035
Urine in ICU, mL^f^	3,521.54 ± 934.20	3,463.49 ± 901.13	3,583.99 ± 966.11	0.171

a n (%); Mean ± SD; median (IQR).

b p-values calculated using an Independent Sample *t*-test for continuous variables and Pearson’s Chi-Square test or Fisher's exact test for categorical variables.

c Mean blood pressure in postoperative time until extubation.

d Mean chest drainage in first 24 hours in the ICU.

e The units of PRBC transfused during ICU and OR.

f The amount of urine in first 24 hours in the ICU. BMI, body mass index; BP, blood pressure; CABG, coronary artery bypass grafting; CAG, coronary angiography; COPD, chronic obstructive pulmonary disease; CPB, cardiopulmonary bypass; Cr, creatinine; EuroSCORE II, European System for Cardiac Operative Risk Evaluation II; HCT, hematocrit; ICU, intensive care unit; LVEF, left ventricular ejection fraction; OR, operating room; PRBC, packed red blood cells; Pre-op, preoperative; Post-op, postoperative; SVD, single-vessel disease; 2VD, two-vessel disease; 3VD, three-vessel disease.

Baseline creatinine levels, body mass index (BMI), hypertension, and EuroSCORE II exhibited no statistically significant differences between the two groups (p = 0.79, p = 0.82, p = 0.06, and p = 0.06, respectively). Conversely, significant inter-group variations were observed in gender distribution, history of chronic obstructive pulmonary disease (COPD), ejection fraction, and incidence of diabetes mellitus (p ≤ 0.01, 0.02, 0.01, and 0.02, respectively). Isolated CABG was the most common surgical procedure, accounting for 78.7% of all cases. The remaining patients underwent combined procedures, including aortic valve replacement (AVR), mitral valve repair, or tricuspid valve repair in conjunction with CABG. There was no statistically significant difference between the two groups with respect to the distribution of surgical types (p = 0.78). The majority of procedures were performed electively (92.7%), with no significant intergroup difference in elective case frequency (p = 0.38).

No statistically significant differences were observed between the two study groups regarding cardiopulmonary bypass duration and aortic cross-clamp time (p = 0.11 and p = 0.22, respectively). The median number of PRBC units transfused in the operating room and during the initial six days of ICU stay was 2.0 (1.0–2.0). Despite a statistically significant difference in the number of PRBC units transfused between the two groups (p < 0.01), the observed variation does not appear to be clinically meaningful. There were no statistically significant differences between the groups regarding intraoperative or postoperative bleeding within the ICU (p = 0.43). Furthermore, comparative analyses revealed no significant differences in the administered doses of protamine (p = 0.19), tranexamic acid (p = 0.65), or heparin (p = 0.21) between the cohorts (Table 1).

Table 2 details patients’ outcomes in each group. With respect to the primary outcomes, neither intubation time nor ICU length of stay differed significantly between the groups (p = 0.21 and p = 0.24, respectively). Although hospital length of stay was initially longer in the ΔHCT ≥ 40% group (p = 0.02), the difference was no longer statistically significant after adjustment. Conversely, although the 30-day mortality difference did not reach statistical significance (p = 0.29), it may nonetheless be of clinical importance. Consistent with these findings, adjusted regression analyses showed no significant association between ΔHCT and any of the primary outcomes (Table 3).

**Table 2:** Comparison of postoperative clinical outcomes between patients with relative HCT reduction ≥ 40% and < 40%.

Variables	Overall (n = 629)^a^	Relative HCT Reduction		p-value^b^
		≥ 40 (n = 326)^a^	< 40 (n = 303)^a^	
**Primary outcomes**				
Mortality, n (%)	8 (1.3%)	6 (1.8%)	2 (0.7%)	0.289
LOS ICU, hours	65.0 (26.0, 97.0)	66.0 (40.0, 109.0)	64.0 (24.0, 96.0)	0.236
Intubation time, hours	16.0 (12.0, 22.0)	16.0 (12.0, 22.0)	16.0 (12.0, 22.0)	0.214
LOS hospital, days	7.0 (6.0, 10.0)	7.0 (6.0, 10.0)	7.0 (5.0, 9.0)	0.025
**Secondary outcomes**				
Composite, n (%)	164 (26.1%)	84 (25.8%)	80 (26.4%)	0.856
Infection, n (%)	53 (8.4%)	25 (7.7%)	28 (9.2%)	0.478
Renal failure^c^, n (%)	12 (1.9%)	9 (2.8%)	3 (1.0%)	0.105
Reoperation, n (%)	113 (18.0%)	60 (18.4%)	53 (17.5%)	0.766
Tamponade, n (%)	38 (6.0%)	16 (4.9%)	22 (7.3%)	0.216
Post-op AF, n (%)	126 (20.0%)	73 (22.4%)	53 (17.5%)	0.125
Post-op encephalopathy, n (%)	57 (9.1%)	28 (8.6%)	29 (9.6%)	0.668
Post-op AKI, n (%)	65 (10.3%)	34 (10.4%)	31 (10.2%)	0.935
Post-op Creatinine, mg.dL^−1^	1.0 (0.8, 1.2)	1.0 (0.8, 1.2)	1.0 (0.9, 1.2)	0.598
Relative HCT reduction (ΔHCT), %	40.02 ± 7.74	45.73 ± 4.60	33.87 ± 5.36	< 0.001
Absolute HCT change, percentage points	-15.65 ± 3.86	-18.20 ± 2.89	-12.90 ± 2.71	< 0.001
Absolute pre- to post-transfusion HCT difference, percentage points	5.22 ± 3.33	6.35 ± 3.36	4.00 ± 2.84	< 0.001
Post-transfusion HCT, %	28.43 ± 3.32	27.88 ± 3.34	29.02 ± 3.20	< 0.001

a n (%); Mean ± SD; median (IQR).

b p-values calculated using an independent *t*-test for continuous variables and Chi-Square test or Fisher's exact test for categorical variables.

c Two patients in the Relative HCT reduction ≥ 40% group received dialysis. Absolute HCT change, absolute difference (percentage points) between baseline and transfusion-time HCT; HCT, hematocrit; ICU, intensive care unit, Intubation time: time from ICU admission to tracheal tube extubation; LOS hospital, time from surgery to hospital discharge, LOS ICU, length of stay in intensive care unit, Post-op, postoperative, Post-op AF, postoperative atrial fibrillation, Post-op AKI: postoperative acute kidney injury, Post-op HCT, postoperative hematocrit, Relative HCT Reduction: relative reduction (%) in HCT from baseline to transfusion time.

**Table 3:** Unadjusted and inverse probability weighting-adjusted linear regression analyses of the association between relative hematocrit reduction and primary outcomes.

Outcomes	Unadjusted			Adjusted		
	Beta	95% CI	p-value	Beta	95% CI	p-value
Log hospital LOS	-0.08	-0.15, -0.01	**0.018**	-0.06	-0.13, 0.00	0.320
Log ICU LOS	-0.11	-0.23, 0.02	0.099	-0.06	-0.18, 0.07	0.392
Intubation time	-3.1	-9.4, 3.3	0.342	-0.82	-6.6, 4.9	0.779

Thirty-day mortality was not included in the regression analyses because only 8 deaths occurred during the study period

CI, confidence interval; ICU, intensive care unit; Intubation time, time from ICU admission to tracheal tube extubation; Log, logarithm; LOS hospital, length of stay in hospital; LOS ICU, length of stay in intensive care unit.

The composite secondary outcomes did not differ significantly between the two groups in unadjusted analysis (p = 0.82). Consistent with these findings, adjusted logistic regression analysis demonstrated no significant association between ΔHCT and the composite secondary outcome (OR = 1.07; 95% CI 0.74–1.55; p = 0.71) (Table 4).

**Table 4:** Unadjusted and IPW-adjusted associations between relative hematocrit reduction and secondary postoperative outcomes.

Outcome	Unadjusted			Adjusted		
	OR	95% CI	p-value	OR	95% CI	p-value
Composite of secondary outcomes	0.96	0.67, 1.38	0.825	1.07	0.74, 1.55	0.707

Composite secondary outcomes include the incidence of infections, tamponade, renal failure, and reoperation due to bleeding.

CI, confidence interval; IPW, inverse probability weighting; OR, odds ratio.

Additionally, the incidence of postoperative encephalopathy, post-op AF and AKI did not differ significantly between the two study groups (p = 0.67, p = 0.12, and p = 0.93, respectively).

Sensitivity analyses yielded findings consistent with the primary analyses, with no significant associations observed between ΔHCT and hospital length of stay, ICU length of stay, or intubation time (Supplementary Table S1).

## Discussion

In this retrospective, single-center, and observational study, patients who received packed red blood cells were analyzed. They were subsequently categorized into two groups based on the relative change in hematocrit (ΔHCT) to compare early postoperative outcomes. Using a restricted cubic spline model, a 40% decline in hematocrit was identified as a potential threshold, dividing patients into two cohorts: those with ΔHCT < 40% and those with ΔHCT ≥ 40. Our findings showed no statistically significant differences in key early postoperative outcomes, including duration of intubation, ICU stay, and overall hospital stay, between the two groups after adjustment for confounders. Secondary outcomes, including the incidence of AF, infection, reoperation due to surgical bleeding, and elevated serum creatinine, were also similar across cohorts. These results suggest that the magnitude of ΔHCT observed at the time of transfusion was not independently associated with early postoperative outcomes, and that other factors, such as surgical complexity, hemodynamic management, and comorbidities, may play a more dominant role in determining outcomes.

The concept of relative hematocrit change remains elusive and appears to be grounded in the notion that both gross and subcellular adaptations can substantially influence physiological responses to changes in hematocrit. Anemia results in a form of hypoxemia known as anemic hypoxia. In response to hypoxia, the body activates several physiological adaptations to preserve tissue oxygenation, including an increase in cardiac output and an enhanced oxygen extraction ratio by organ tissues. In contrast, it is believed that there are no comparable compensatory mechanisms ‒ such as the ability to significantly elevate hemoglobin concentration or oxygen saturation beyond normal physiological limits ‒ to mitigate the reduction in oxygen delivery associated with decreased cardiac output.^1^

Most recent studies have focused on comparing restrictive versus liberal blood transfusion strategies and their impact on clinical outcomes. Although findings have been somewhat inconsistent, the overall body of evidence tends to support restrictive transfusion approaches, given their association with lower mortality rates and reduced postoperative complications.^13^, ^14^, ^15^ However, relatively very few studies have evaluated the impact of changes in hemoglobin levels (ΔHb) on postoperative outcomes in surgical patients. In a retrospective study, Spolverato G and colleagues reviewed mortality, ischemia-specific complications, and ΔHb in patients undergoing major gastrointestinal (GI) surgery.^6^ In contrast to our study, they found that a decrease in Hb ≥ 50%, independent of whether the nadir Hb remained ≥ 7 g.dL^−1^, was significantly associated with increased postoperative morbidity, including ischemic events. Their findings highlighted that the magnitude of relative hemoglobin decline, not just the absolute nadir hemoglobin value, plays a critical role in predicting adverse outcomes, suggesting that current transfusion practices that rely solely on nadir thresholds may overlook clinically relevant anemia-related risks.

Another study investigated the relationship between the degree of hemoglobin decline and clinical outcomes ‒ specifically mortality and the need for urgent therapeutic endoscopy ‒ in patients with GI bleeding.^7^ Nadir hemoglobin alone was not significantly associated with adverse outcomes such as in-hospital mortality or urgent endoscopy. However, a relative hemoglobin drop of 30%–40%, combined with moderate nadir Hb levels (8–9 g.dL^−1^), was associated with higher adverse event rates. Among transfused patients, outcomes were worse when hemoglobin drop was substantial, regardless of nadir hemoglobin levels.

In a recent retrospective observational study, Lei Du and colleagues examined the association between varying degrees of hemoglobin change (ΔHb) and early postoperative outcomes in patients undergoing cardiac surgery.^16^ The study demonstrated a non-linear association between ΔHb and adverse outcomes, modulated by preoperative anemia status and transfusion volume. In anemic patients, a ΔHb ≥ 30% was linked to increased risk; in non-anemic patients, risk increased at ΔHb ≥ 50%. Transfusion effects varied: in non-anemic patients, small transfusions were protective at high ΔHb, while large transfusions increased risk regardless of ΔHb. In anemic patients, small transfusions reduced risk at lower ΔHb, but only high-volume transfusions increased risk at higher ΔHb. While the majority of patients in that study underwent valvular surgery, our investigation focused primarily on individuals undergoing CABG, with most receiving isolated CABG procedures. It is reasonable to assume that more complex surgical procedures and greater blood loss necessitate higher transfusion volumes, which may predispose to hemodynamic instability and adverse outcomes. By narrowing our cohort to a less complex surgical population with reduced procedural complexity and heterogeneity, we aimed to minimize confounding. We hypothesize that, in routine clinical practice, most transfusions are not massive. Supporting this, most randomized controlled trials (RCTs) evaluating transfusion thresholds have employed a one-unit transfusion strategy and demonstrated no additional benefit from administering more blood than necessary.^17^ Furthermore, to eliminate the confounding influence of cardiopulmonary bypass, patients undergoing off-pump procedures were excluded from the analysis. In addition, we used the EuroSCORE II system to enhance group comparability by accounting for overall clinical status and comorbidities, including variables such as ejection fraction, surgical risk, pulmonary hypertension, and other key factors.^18^

The Lei et al. study categorized patients based on the presence of preexisting anemia but did not clearly differentiate between its chronic and acute forms. In contrast, we excluded individuals with recent transfusions or ongoing anemia treatment, thereby minimizing confounding factors that could influence physiological responses. Transfusion thresholds were similar between studies, with red blood cell transfusions initiated at an HCT of ≤ 21% during cardiopulmonary bypass and 21%–24% throughout the perioperative period. While the prior investigation examined hemoglobin declines of 30% and 50%, we employed a 40% ΔHCT threshold for cohort stratification. Intergroup differences in the earlier study were primarily observed in the context of significant drops in hemoglobin and increased transfusion rates. However, critical clinical data such as bleeding volume, hemodynamic stability, and surgical complexity were not reported, despite their relevance to transfusion requirements. On the contrary, our study found no significant differences in blood drainage volumes in the operating room or ICU between Group A and Group B (p = 0.427). Moreover, perioperative PRBC usage in our study was lower than the one reported in the previous study.

Our primary outcomes included early postoperative parameters ‒ duration of intubation, ICU stay, and total hospital stay ‒ which were not evaluated in the earlier study. After adjustment for ΔHCT, no significant differences were observed between groups for these outcomes. Secondary endpoints such as incidence of AF, reoperation due to surgical bleeding, postoperative encephalopathy, and AKI were likewise comparable. These findings suggest that postoperative outcomes may be more closely associated with factors reflecting clinical severity, particularly hypoxemic hypoxia secondary to circulatory dysfunction, than with the magnitude of hematocrit decline observed at the time of transfusion.

Although the observed difference in mortality (1.8% vs. 0.7%) did not reach statistical significance (p = 0.29), the absolute difference may still be clinically meaningful and warrants further investigation in larger, well-powered studies.

### Study limitations

This study has several limitations that should be acknowledged. First, its retrospective design inherently limits the ability to establish causality and is subject to potential selection bias and unmeasured confounding. Second, the relatively small sample size and single-center setting may limit the generalizability of our findings; this limitation is partly due to the specific focus of our study population, as we included only patients who underwent CABG, although a very small subset also underwent combined valve procedures. Third, the database lacked certain physiologic parameters ‒ such as arterial blood gas indices and central or mixed venous oxygen saturation (ScvO_2_/SvO_2_) ‒ which would have enabled a more comprehensive assessment of tissue oxygenation and oxygen extraction ratios, providing further insight.

## Conclusion

In this retrospective cohort study, postoperative outcomes did not significantly differ between patients with a relative hematocrit decline of ≥ 40% and those with a decline of < 40% at the time of transfusion. A relative decline of 40% or more at the time of transfusion was not associated with differences in early outcomes after adjustment. These findings indicate that the degree of hematocrit reduction preceding transfusion may not be independently associated with early postoperative recovery following CABG. Rather, factors such as surgical complexity, perioperative hemodynamic stability, and underlying patient comorbidities may play a more pivotal role in determining clinical outcomes. These observations underscore the need for further studies to determine the clinical relevance of a relative hematocrit decline at the time of transfusion and its relationship to postoperative outcomes following CABG.

## AI assistance disclosure

The authors did not use generative artificial intelligence in the preparation of this manuscript.

## Data availability statement

The datasets generated and/or analyzed during the current study are available from the corresponding author upon reasonable request.

## Authors’ contributions

Fatemeh Sadr: Data collection; Literature review.

Arash Jalali: Statistical analysis; Interpretation of results, and critical revision of the manuscript.

Ahmad Vakili-Basir: Statistical analysis; Visualization; Designed and created all tables and figures.

Mina Pashang: Data collection.

Khosro Barkhordari: Conceptualization; Supervision; Project administration; Manuscript revision.

## Conflicts of interest

The authors declare no conflicts of interest.

## Appendix Supplementary materials

Image, application 1

## Footnotes

Supplementary material associated with this article can be found in the online version at doi:10.1016/j.bjane.2026.844813.
