# Supplementary material for: Relative hematocrit declines at the time of transfusion and postoperative outcomes after coronary artery bypass grafting: a retrospective cohort study
Source: Braz J Anesthesiol. 2026 Aug 7;76(5):844813. doi: 10.1016/j.bjane.2026.844813 (PMC13594730; doi:10.1016/j.bjane.2026.844813)
Supplement: Supplementary file 1 [file mmc1.docx]

**BJAN-D-25-00538_ Supplementary Material**

**Supplementary Table S1** Sensitivity analysis for missing data.

| **Outcomes** | **Sensitivity Analysis** | | |
| --- | --- | --- | --- |
|  | **Beta** | **95% CI** | **p-value** |
| Log LOS hospital | -0.04 | -0.10, 0.02 | 0.209 |
| Log LOS ICU | -0.04 | -0.17, 0.08 | 0.507 |
| Intubation time | -1.3 | -6.3, 4.1 | 0.629 |

CI, confidence interval; ICU, intensive care unit, Intubation time: time from ICU admission to tracheal tube extubation; Log, logarithm; LOS hospital, length of stay in hospital; LOS ICU, length of stay in intensive care unit.

**Supplementary Figure S1** Histogram showing the distribution of stabilized inverse Probability Weights (sIPW) from the propensity score model. Most weights are clustered around 1.0 (median = 0.80), illustrating the variability in weighting across participants and balanced weighting.

**Supplementary** **Figure S2** Assessment of covariate balance before and after inverse probability weighting. Absolute standardized mean differences are shown for each covariate, with the orange line representing the original (unweighted) sample and the blue line representing the weighted sample. Values closer to zero indicate improved balance, and the vertical dashed line at 0.1 indicates the commonly accepted threshold for adequate balance. AKI, acute kidney injury; BMI, body mass index; COPD, chronic obstructive pulmonary disease; CS, cigarette smoking history; Diff HCT, difference between baseline and transfusion-time hematocrit; DM, diabetes mellitus, duration of perfusion time: cardiopulmonary bypass duration; EF, ejection fraction; HLP, hyperlipidemia; OR, operation room; Status procedure: number of coronary vessels undergoing bypass grafting; uni.PC, packed red blood cell unit.
